# Transcription Analysis of the *THBS2* Gene through Regulation by Potential Noncoding Diagnostic Biomarkers and Oncogenes of Gastric Cancer in the ECM-Receptor Interaction Signaling Pathway: Integrated System Biology and Experimental Investigation

**DOI:** 10.1155/2023/5583231

**Published:** 2023-12-22

**Authors:** Ali Barani, Kamyar Beikverdi, Benyamin Mashhadi, Naeimeh Parsapour, Mohammad Rezaei, Pegah Javid, Mansoureh Azadeh

**Affiliations:** ^1^Zist Fanavari Novin Biotechnology Institute, Isfahan, Iran; ^2^Department of Biosciences, University of Milan, Milan, Italy; ^3^Department of Molecular Medicine, University of Pavia, Pavia, Italy; ^4^Institute of Biochemistry and Biophysics, University of Tehran, Tehran, Iran; ^5^Department of Immunology, Genetics and Pathology, Faculty of Medicine, Uppsala University, Uppsala, Sweden; ^6^Department of Biology and Biotechnology, University of Pavia, Pavia, Italy; ^7^Molecular Genetics Research Lab, Persian Gulf Biotechnology Park, Qeshm Island, Hormozgan, Iran

## Abstract

**Background:**

Gastric cancer (GC) is the second most frequent cause of cancer-related death worldwide and the fourth most common malignancy. Despite significant improvements in patient survival over the past few decades, the prognosis for patients with GC remains dismal because of the high recurrence rate. In this comprehensive system biology and experimental investigation, we aimed to find new novel diagnostic biomarkers of GC through a regulatory RNA interaction network.

**Methods:**

Gene expression, coexpression, and survival analyses were performed using microarray and RNAseq datasets (analyzed by RStudio, GEPIA2, and ENCORI). RNA interaction analysis was performed using miRWalk and ENCORI online databases. Gene set enrichment analysis (GSEA) was performed to find related signaling pathways of up- and downregulated genes in the microarray dataset. Gene ontology and pathway enrichment analysis were performed by the enrichr database. Protein interaction analysis was performed by STRING online database. Validation of expression and coexpression analyses was performed using a qRT-PCR experiment.

**Results:**

Based on bioinformatics analyses, *THBS2* (FC: 7.14, FDR < 0.0001) has a significantly high expression in GC samples. lncRNAs *BAIAP2-AS1*, *TSIX*, and *LINC01215* have RNA interaction with *THBS2*. *BAIAP2-AS1* (FC: 1.44, FDR: 0.018), *TSIX* (FC: 1.34, FDR: 0.038), and *LINC01215* (FC: 1.19, FDR: 0.046) have significant upregulation in GC samples. *THBS2* has a significant role in the regulation of the ECM-receptor signaling pathway. miR-4677-5p has a significant RNA interaction with *THBS2*. The expression level of *THBS2*, *BAIAP2-AS1*, *TSIX*, and *LINC01215* has a nonsignificant negative correlation with the survival rate of GC patients (HR: 0.28, logrank *p*: 0.28). qRT-PCR experiment validates mentioned bioinformatics expression analyses. *BAIAP2-AS1* (AUC: 0.7136, *p* value: 0.0096), *TSIX* (AUC: 0.7456, *p* value: 0.0029), and *LINC01215* (AUC: 0.7872, *p* value: 0.0005) could be acceptable diagnostic biomarkers of GC.

**Conclusion:**

*BAIAP2-AS1*, lncRNA *LINC01215*, lncRNA *TSIX*, and miR-4677-5p might modulate the ECM-receptor signaling pathway via regulation of *THBS2* expression level, as the high-expressed noncoding RNAs in GC. Furthermore, mentioned lncRNAs could be considered potential diagnostic biomarkers of GC.

## 1. Introduction

Globally, gastric cancer (GC) is the second most common cause of cancer-related mortality and the fourth most prevalent malignant disease [1]. The prognosis for patients with GC is still poor because of the high recurrence rate, despite major advancements in patient survival over the past several decades [2]. GC is frequently identified at an advanced stage. Since most cases of GC are asymptomatic until they reach late stages, it is crucial to use efficient screening techniques to identify cases early in order to reduce GC fatalities [2]. In order to serve as a marker for healthy biologic processes, destructive processes, or pharmacological responses to therapeutic interventions, biomarkers are traits that can be objectively studied and measured. Recent developments in genome analysis have led to the discovery of several biomarkers relating to DNA, RNA, exosomes, etc. The creation of these biomarkers in the field of cancer therapy is anticipated to have a significant impact on the progression of the disease, the choice of effective therapeutic approaches, and effective follow-up programs [2].

Better technology and bioinformatics analyses to comprehend dynamic changes in biology and tumor plasticity will be linked to further advancements in cancer therapy. Consideration must be given to tumor heterogeneity, the interaction between the cancer genome and the epigenome, the surrounding microenvironment, and vertical access (changes over time) of cancer biological components to address molecular evolution and horizontal access (changes over sites of disease involvement) to address tumor heterogeneity. The potential of computational medicine and data sharing inspires researchers to create exciting initiatives that integrate big data and bioinformatics. The possibility of treating cancer ultimately rests with the development of efficient treatment approaches, well-planned clinical trials, and coordinated efforts among crucial players in cancer therapy [3].

Long noncoding RNAs (lncRNAs) have gotten a lot of attention as possible diagnostic, prognostic, or predictive biomarkers because of their high specificity and ease of accessibility in a noninvasive way, as well as their aberrant expression under diverse pathological and physiological situations. They could possibly be used as stomach cancer treatment targets [4].

Based on previous studies, lncRNAs have significant roles in the different biological processes correlated to GC. For example, lncRNA *PCAT-1*, which is significantly expressed in tissues and cells of gastric cancer resistant to DDP, increases DDP resistance in gastric cancer cells by engaging *EZH2* to epigenetically repress *PTEN* expression and controlling the *miR-128/ZEB1* axis [5, 6]. EZH2 is also considered as a potential prognostic biomarker of hepatocellular carcinoma [7]. Similarly, it has been discovered that the DDP-resistant gastric cancer cells SGC7901/DDP and BGC823/DDP express the lncRNA *DANCR* at a high level. *DANCR* knockdown in these cells encourages apoptosis and prevents cell division. On the other hand, DDP-induced SGC901 and BGC823 cells with overexpressed *DANCR* might increase the expression of *MDR* genes *MDR1* and *MRP1* [8]. Through the upregulation of *MDR1*, *MRP1*, and *Bax* expression as well as the downregulation of Bcl-2 expression, lncRNA *SNHG5* decreased the DDP sensitivity of the gastric cancer cells BGC823 and SGC7901 [9].

Based on GeneCards (http://genecards.org), *THBS2* produces a member of the thrombospondin family of proteins. It is a homotrimeric glycoprotein with disulfide links that mediate interactions between cells and between cells and a matrix. It has been demonstrated that this protein acts as a powerful inhibitor of tumor angiogenesis and proliferation. Studies of the mouse equivalent imply that this protein may modify the mesenchymal cells' cell surface characteristics and be involved in cell adhesion and migration. Through regulation by miR-221-3p, *THBS2* might promote angiogenesis in cervical cancer [10]. Zhang et al. in 2022 revealed that *THBS2* has a significant upregulation in gastric cancer patients. Also, this study revealed that high expression of *THBS2* has significant correlations with pathological grade, T stage, and poor overall survival of patients [11].

In this study, we performed a comprehensive bioinformatics investigation and experimental validation to find potential novel biomarkers of GC. Also, we demonstrate novel RNA and protein interaction networks to find novel regulatory noncoding RNAs in GC patients. The central core of this study is the *THBS2* gene as a potential misregulated mRNA in GC patients.

## 2. Materials and Methods

### 2.1. Microarray Data Analysis

Microarray analysis was performed on the gastric cancer-related datasets. GSE54129 was investigated in order to find the differentially expressed genes (DEGs) in the gastric cancer microarray datasets. 111 GC samples and 21 control samples from this dataset were evaluated. GPL570 (HG-U133 Plus 2, Affymetrix Human Genome U133 Plus 2.0 Array) is the source of this dataset. The raw data from the GEO online database (https://www.ncbi.nlm.nih.gov/geo/) was transmitted to the RStudio environment and then normalized using the affy [12] package. The microarray dataset underwent statistical analysis using the limma [13] package. The affy and limma packages were obtained from the Bioconductor online site (https://www.bioconductor.org/). For the analysis of microarray data, a significance threshold of 0.0001 was chosen (adjusted *p* value). The microarray data analysis visualizations were created using the ggplot2 [14] and pheatmap packages, which are available from CRAN (https://cran.r-project.org). In this microarray study, the expression of 47568 RNA transcript (21257 genes) was investigated. Following normalization (RMA method), logarithmic scaling, and elimination of the transcripts with no expression in the dataset, the difference in the expression level of all RNAs was calculated. The RNAs with logFC > 3 were chosen as the upregulated RNAs, while logFC < −3 was selected as the threshold of low expression.

### 2.2. Gene Set Enrichment Analysis (GSEA)

The samples in the GSE54129 dataset were split into control and tumor samples. Gene set enrichment analysis (GSEA) (https://software.broadinstitute.org/gsea/) was used to investigate related signaling pathways [15]. *p* < 0.05 was used to assess what words were significant.

### 2.3. Gene Ontology (GO), Expression, Survival, and RNA Interaction Analyses

ENCORI online database carried out the mRNA-lncRNA interaction analyses (https://starbase.sysu.edu.cn/) [16]. The online software GEPIA2 [17] (http://gepia2.cancer-pku.cn/) and ENCORI carried out expression, correlation, and survival analyses. Using Cytoscape software (version 3.8.2), RNA interactions were visualized [18, 19]. The STRING online database analyzed and visualized protein-protein interactions [20]. GO and pathway enrichment analyses were performed by enrichr online database [21–23]. The investigation of the interactions between microRNAs and mRNAs was carried out by miRWalk (http://mirwalk.umm.uni-heidelberg.de/) [24–26]. The top miRNAs with the following criteria were selected: binding probability, 1; position, 3′UTR (seed region); and lowest binding energy. Sampe size was calculated using the following formula: *n* = ((*σ*_*A*_^2^ + *σ*_*B*_^2^)(*Z*_1−*α*/2_ + *Z*_1−*β*_)^2^)/*δ*^2^. *σ*_*A*_^2^ + *σ*_*B*_^2^ is the variance of control and tumor samples, and *Z*_1−*α*/2_ and *Z*_1−*β*_ are the statistical power of samples (numeric values: 1.96 and 0.84, respectively).

### 2.4. Clinical Characteristics of Tissue Samples

All patients signed written consent forms, and all methods for the research in this study involving human samples were approved by the Al-Zahra Hospital Ethics Committee, Isfahan University of Medical Science. Samples of normal gastric tissue and gastric cancer from 25 individuals with gastric cancer were compared in a case-control study. Normal gastric tissues are adjacent to tumor samples. None of the patients had ever received radiation or chemotherapy. Tissue samples were rinsed in distilled water and promptly frozen in liquid nitrogen for RNA Later solution (Invitrogen, USA) immersion for pathologist assessment. The clinicopathological characteristics of patients with breast and stomach cancer are listed in Table 1.

### 2.5. RNA Extraction, cDNA Synthesis, and qRT-PCR Experiment

TRIzol was employed to extract the RNA from both tumorous and normal tissues (Invitrogen, Carlsbad, CA, USA). Following the RNA extraction steps, cDNA synthesis was performed using the TaKaRa cDNA synthesis kit according to the manufacturer's instructions (TaKaRa, Tokyo, Japan). SYBR green, an Amplicon Company product from Denmark, was utilized to do real-time PCR, and a MIC real-time PCR instrument was used to perform reverse transcription quantitative polymerase chain reaction (RT-qPCR) experiment. Following conducting, the following parameters for the PCR reactions were set: initial denaturation, 95°C for 15 minutes; secondary, 95°C for 15 seconds; 60°C for 20 seconds; and 72°C for 20 seconds. There were 40 cycles in total. The sequences of the primers, which were created by TAQ Copenhagen Company (Denmark), are displayed in Table 1. As an internal control, the related expression was normalized using the quantity of GAPDH. In Table 2, the primer sequence is displayed.

The GraphPad Prism application was used to statistically evaluate the real-time PCR data and related visualizations (version 8). The qRT-PCR data were compared using the CT method to determine the expression levels between the tumor and control samples [27]. The Shapiro-Wilk test was used to the expression data in order to ascertain whether the data were normal. Using paired *t*-test and Wilcoxon test on the CT data, the expression levels in tumor and control samples were compared. The DEG analysis of the microarray data was performed in RStudio (4.1.2). Based on sensitivity and specificity, the recipient operating characteristic (ROC) analysis was carried out by the GraphPad Prism for the real-time PCR datasets. A *p* value of less than 0.05 was selected as the significance threshold for this study. AUC values between 0.7 and 0.8 in the ROC analysis are regarded as acceptable, 0.8 and 0.9 as good (signifying a good biomarker), and 0.9 and 1 as excellent (indicating an outstanding biomarker).

## 3. Results

### 3.1. Microarray Data Analysis

**Figure 1:**
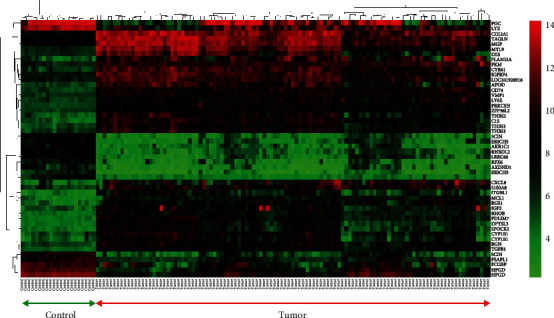
Heatmap of top 50 differentially expressed genes in GSE54129. Genes are categorized into different clusters, based on the expression level. Samples also are categorized into two main clusters based on group (control or tumor). The first 21 columns in the left side of heatmap represent normal samples and other 111 columns in the right side of heatmap are showing the tumor samples.

**Figure 2:**
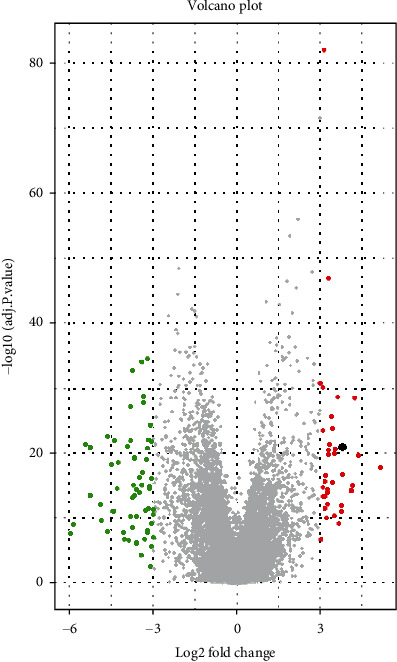
Volcano plot showing the DEGs in the GSE54129. Red color indicates the upregulated genes and green color indicates the downregulated genes. *THBS2* is indicated by a black point in the plot as a significantly upregulated gene. Further analyses were performed on THBS2 as a significant high-expressed mRNA.

Microarray data analysis revealed 37 upregulated genes and 60 low-expressed genes in the GSE54129 dataset. List of top 20 up- and downregulated genes is provided in Table 3.  Figure 1 shows the heatmap of top 50 DEGs. Correlation clustering method was performed on samples and genes in this heatmap. Control and tumor samples are completely separated in different clusters. Also, upregulated and downregulated genes are completely clustered. Volcano plot of all genes in GSE54129 revealed up- and downregulated genes in the dataset (Figure 2).

### 3.2. GSEA

**Figure 3:**
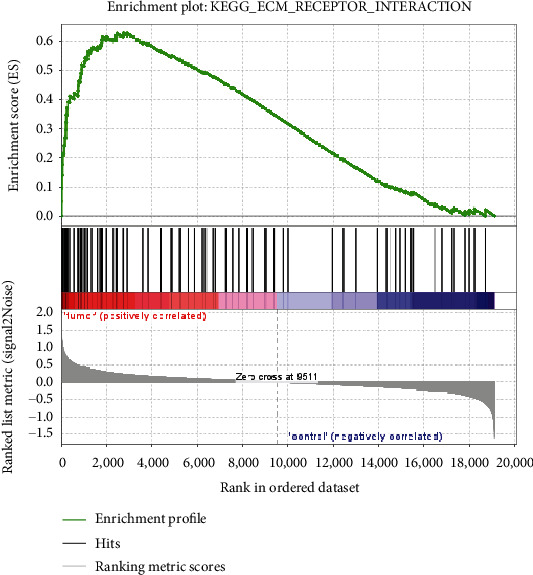
Profile of the running ES score and positions of gene set members on the rank ordered list. Area under the yellow plot indicates the significance level of pathway analysis. Each gene in the ECM-receptor pathway is shown in the plot by a bar line. Genes with higher expression change are located in the left side on the plot. The first gene (the most significant dysregulated gene) is the THBS2 that is located at the left side with higher score.

**Figure 4:**
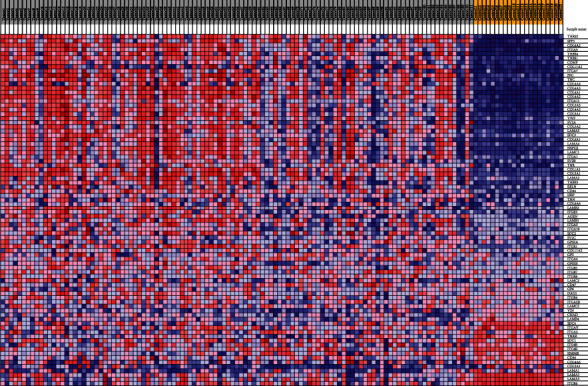
Heatmap of upregulated genes, involved in ECM-receptor signaling pathway. Yellow color indicates normal samples, and the gray color indicates tumor samples. Red color indicates higher expression level of genes in related sample, and the blue color indicates lower expression level. THBS2 has the most change in the expression level, as a significant high-expressed gene in the GC samples.

Based on GSEA, upregulated genes of GSE54129 regulate the ECM-receptor interaction signaling pathway (Figures 3 and 4). Based on mentioned analysis, *THBS2* is the most significant upregulated gene in the ECM-receptor interaction pathway (FWER *p* value < 0.0001, rank metric score: 1.38). *THBS2* was selected for further investigation. List of significant upregulated genes in mentioned signaling pathway is provided in Table 4.

### 3.3. RNA and Protein Interaction Analysis

**Figure 5:**
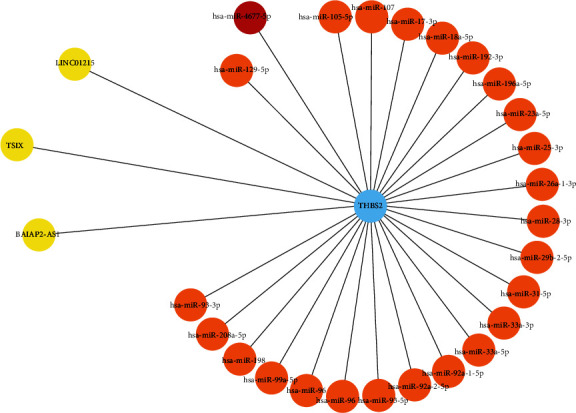
miRNA and lncRNA interaction analysis of *THBS2*. Based on the miRWalk database, miRNA interaction analysis was performed. miR-4677-5p has the strongest interaction with the 3′UTR area of THBS2. In addition, lncRNAs *LINC01215*, *TSIX*, and *BAIAP2-AS1* have direct interaction with *THBS2* mRNA, based on the lncRNA interaction analysis using lncRRIsearch database. However, there is no interaction between miRNAs and lncRNAs in this network.

**Figure 6:**
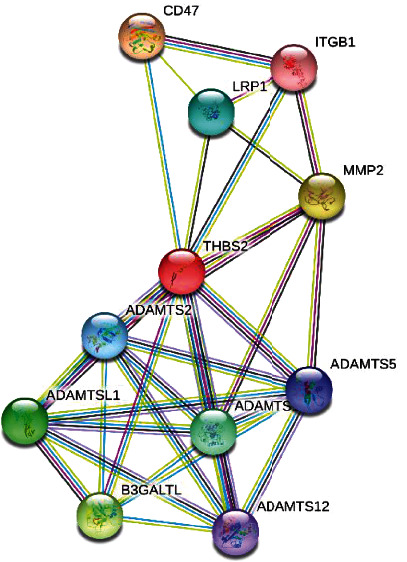
Protein-protein interaction analysis of *THBS2* by STRING online database.

lncRNA-mRNA interaction analysis by lncRRIsearch database revealed that *THBS2* has significant interaction with *LINC01215*, lncRNA *TSIX*, and lncRNA *BAIAP2-AS1*. lncRRIsearch finds physical interaction of lncRNAs and mRNAs. Based on this result, three mentioned lncRNAs could regulate the activity and expression level of *THBS2* through physical interaction with mRNA *THBS2*. Also, miRNA interaction analysis revealed that *THBS2* has a significant interaction with miR-4677-5p (score (binding probability): 1, energy: -22.5, Figure 5). Based on this information, miR-4677-5p could suppress the expression level of THBS2 through direct interaction with the 3′UTR region of mRNA *THBS2*. Protein-protein interaction analysis revealed that *THBS2* protein has significant protein interaction with following proteins: *ADAMTS1*, *ADAMTS12*, *ADAMTS2*, *ADAMTS5*, *ADAMTSL1*, *B3GALTL*, *CD47*, *ITGB1*, *LRP1*, and *MMP2* (Figure 6).

### 3.4. GO and Pathway Enrichment Analyses

Pathway enrichment and GO analyses were performed on mentioned proteins to find the biological processes, molecular functions, and cellular component, related to *THBS2* and its interactome. Based on mentioned analyses, *THBS2* and its interactome are located in collagen-containing extracellular matrix (GO:0062023). Also, mentioned proteins (Figure 6) mostly regulate metalloendopeptidase activity (GO:0004222). Furthermore, mentioned genes are significantly involved in extracellular structure organization (GO:0043062) process (Table 5). Pathway enrichment analysis revealed that *THBS2* is significantly regulated following signaling pathways: ECM-receptor interaction, malaria, leukocyte transendothelial migration, phagosome, focal adhesion, and proteoglycans in cancer (Table 6).

### 3.5. Coexpression Analysis of *THBS2* with lncRNAs

**Figure 7:**
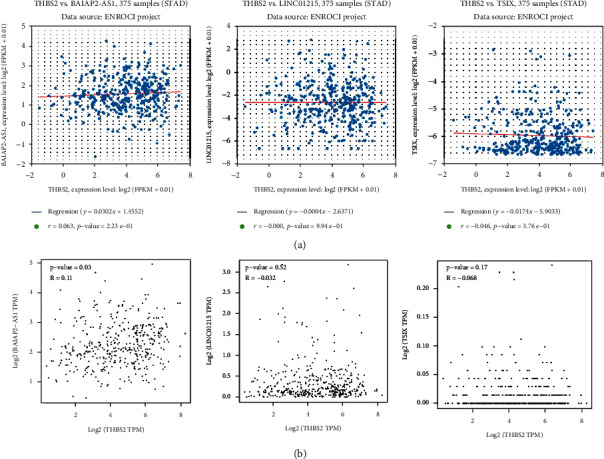
Coexpression analysis of *THBS2* and lncRNAs, based on ENCORI and GEPIA2 online databases. (a) Coexpression analysis based on ENCORI revealed that *THBS2* has no significant coexpression with BAIAP-AS1, *TSIX*, and *LINC01215*. (b) Coexpression analysis based on GEPIA2 revealed that *THBS2* has a significant coexpression with *BAIAP2-AS1*.

Coexpression analysis of *THBS2* and lncRNAs with ENCORI revealed that *THBS2* has no significant coexpression with lncRNA *BAIAP2-AS1* (*r*: 0.063, *p* value: 2.23*E*‐01), *LINC01215* (*r*: 0.000, *p* value: 9.94*E*‐01), and *TSIX* (*r*: -0.046, *p* value: 3.76*E*‐01). However, same analyses by GEPIA2 revealed that *THBS2* expression has a significant slight positive correlation with *BAIAP2-AS1* (*r*: 0.11, *p* value: 0.03, Figure 7). However, due to the low *r*-value of this correlation, demonstrated correlation result needs more validations.

### 3.6. *THBS2* and lncRNAs Have Significant Upregulation in the GC Samples

**Figure 8:**
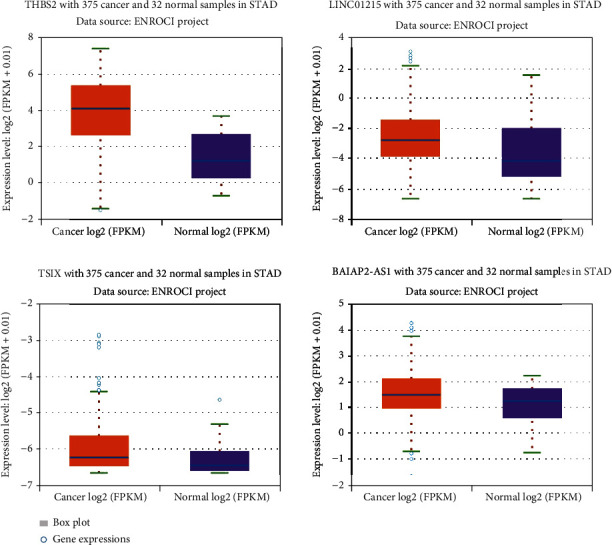
Relative expression analysis of *THBS2* and selected lncRNAs, based on ENCORI online database. Based on expression analysis by ENCORI, *THBS2*, *LINC01215*, *BAIAP2-AS1*, and *TSIX* have significant high expression in GC samples, compared to control.

**Figure 9:**
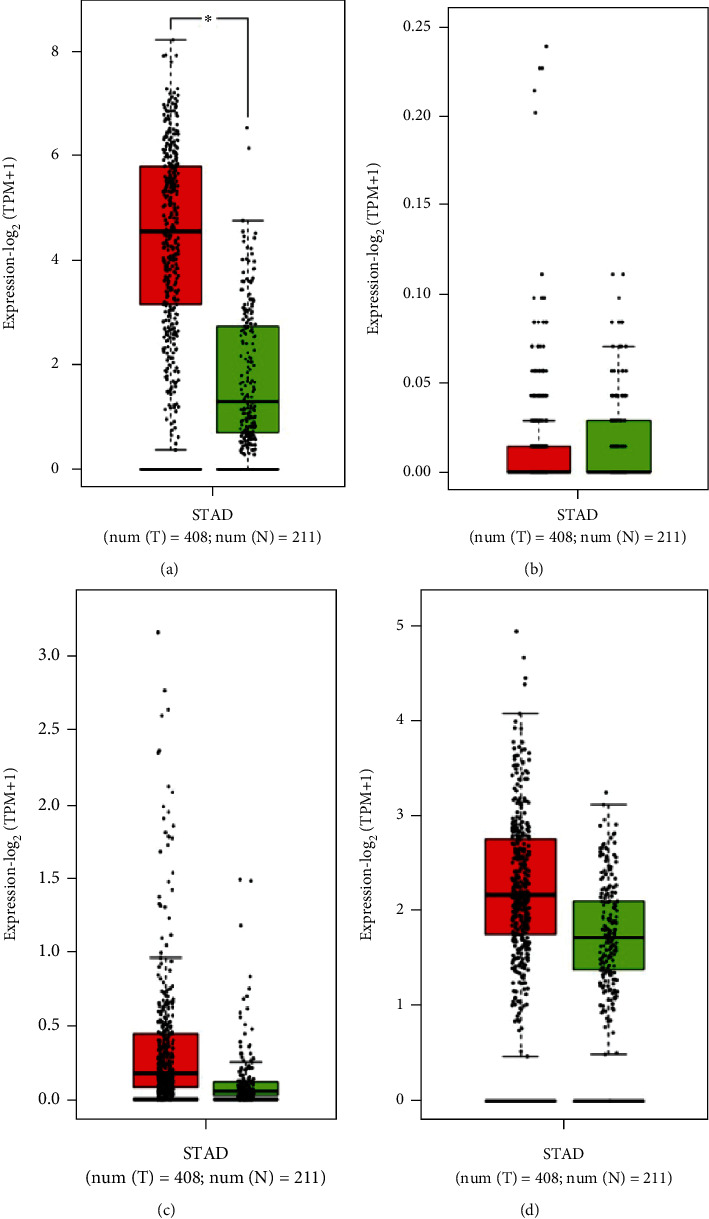
Relative expression analysis of *THBS2* (a), *TSIX* (b), *LINC01215* (c), and *BAIAP2-AS1* (d). Based on GEPIA2 online database, THBS2 has a significant high expression in GC samples. Based on this database, *LINC01215*, *BAIAP2-AS1*, and *TSIX* have no significant change in the GC samples.

**Figure 10:**
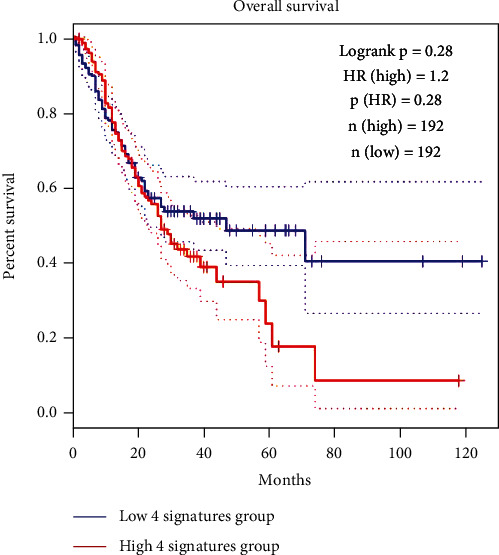
Survival analysis of *THBS2*, *BAIAP2-AS1*, *LINC01215*, and *TSIX*. Based on this analysis, mentioned RNAs have no significant correlation with the low survival rate of GC patients. This analysis was performed using GEPIA2 online database, and the statistical parameter was based on the default setting of this database.

Expression analysis of *THBS2* and selected lncRNAs was performed by GEPIA2 and ENCORI online databases. Based on mentioned analyses, *THBS2* (FC: 7.14, FDR < 0.0001), *BAIAP2-AS1* (FC: 1.44, FDR: 0.018), *TSIX* (FC: 1.34, FDR: 0.038), and *LINC01215* (FC: 1.19, FDR: 0.046) have significant upregulation in GC samples, compared to control (Figures 8 and 9). Furthermore, survival analysis revealed that high expression of *THBS2*, *LINC01215*, *TSIX*, and *BAIAP2-AS1* has a nonsignificant correlation with low survival rate of GC patients (HR: 0.28, logrank *p*: 0.28, Figure 10).

### 3.7. qRT-PCR Data Analysis

**Figure 11:**
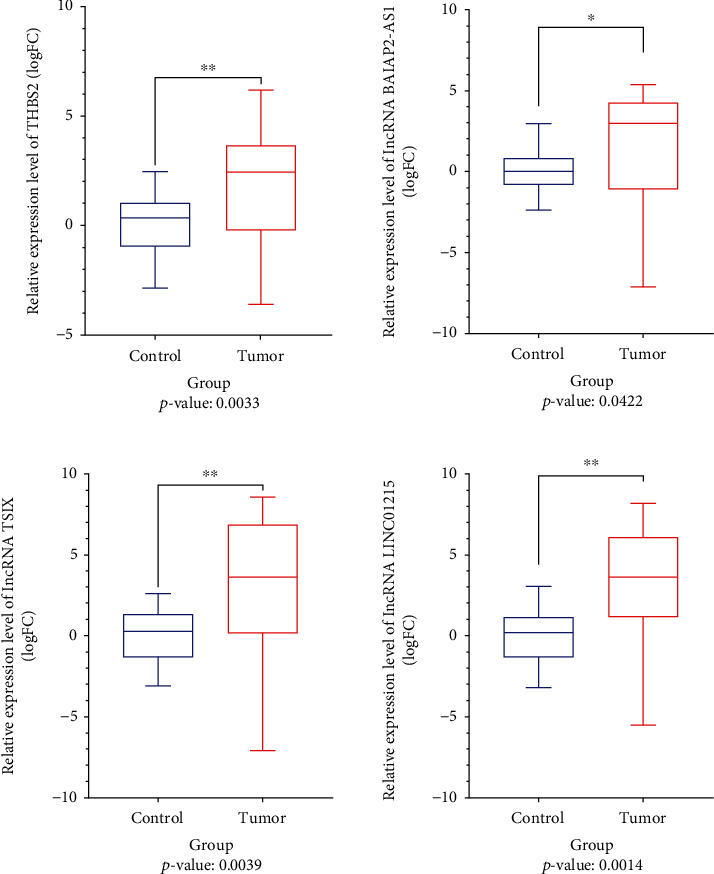
qRT-PCR data analysis was performed using GraphPad Prism software. All statistical tests and graphs of qRT-PCR experiment were performed and visualized using that software. Relative expression analysis of qRT-PCR data revealed that *THBS2*, *BAIAP2-AS1*, *TSIX*, and *LINC01215* have significant upregulation in GC samples, compared to control.

**Figure 12:**
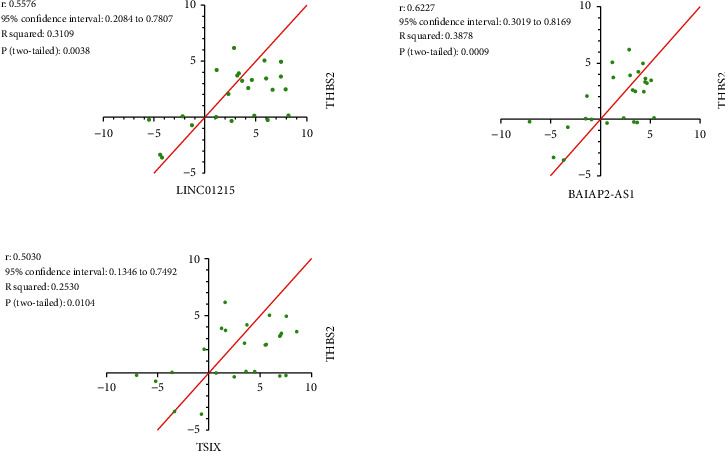
Spearman correlation analysis was performed using GraphPad Prism. Spearman correlation analysis revealed that *THBS2* has significant positive coexpression with lncRNAs *BAIAP2-AS1*, *LINC01215*, and *TSIX*.

**Figure 13:**
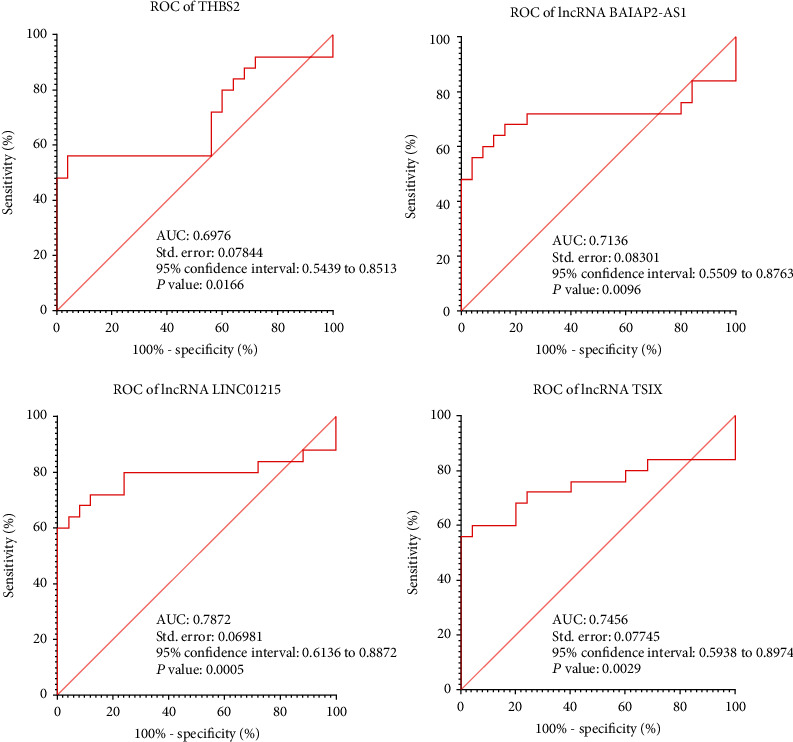
ROC analysis revealed that *BAIAP2-AS1*, *LINC01215*, and *TSIX* could be considered as the potential diagnostic biomarkers of gastric cancer.

For the validation of mentioned results, qRT-PCR experiment was performed. Based on mentioned analysis, *THBS2* (logFC: 1.719, *p* value: 0.0033), *BAIAP2-AS1* (logFC: 3.495, *p* value: 0.0422), *TSIX* (logFC: 2.821, *p* value: 0.0039), and *LINC01215* (logFC: 3.119, *p* value: 0.0014) have significant high expression in human GC samples, compared to control (Figure 11). Based on the Spearman correlation analysis, *THBS2* has significant positive coexpression with *LINC01215* (*r*: 0.5576, *p* value: 0.0038), *TSIX* (*r*: 0.5030, *p* value: 0.0104), and *BAIAP2-AS1* (*r*: 0.6227, *p* value: 0.0009, Figure 12). ROC analysis revealed that *BAIAP2-AS1* (AUC: 0.7136, *p* value: 0.0096), *TSIX* (AUC: 0.7456, *p* value: 0.0029), and *LINC01215* (AUC: 0.7872, *p* value: 0.0005) could be acceptable diagnostic biomarkers of GC (Figure 13).

## 4. Discussion

Our study demonstrates comprehensive novel results about the possible roles of coding and noncoding RNAs in GC development. Based on our investigation, lncRNAs *BAIAP2-AS1*, *LINC01215*, and *TSIX* could be considered novel potential diagnostic biomarkers of GC. Based on our bioinformatics and experimental analyses, mentioned lncRNAs might regulate the expression level of *THBS2*, a high-expressed mRNA, in GC patients. *THBS2* and its interactome regulate the ECM-receptor interaction signaling pathway. Previous studies approved the possible role of the ECM-receptor signaling pathway in the regulation of progression, survival rate, and tumorigenesis of GC [28]. Based on our investigation, *BAIAP2-AS1*, *LINC01215*, and *TSIX* might regulate the ECM-receptor signaling pathway via regulation of the *THBS2* signaling pathway. There was no previous study about the possible regulatory role of mentioned lncRNAs in the ECM-receptor signaling pathway. High expression of mentioned lncRNAs might disturb normal processes of the ECM-receptor signaling pathway. This disturbance may lead the normal gastric cells to malignancy. Furthermore, based on our analyses, the expression level of *THBS2*, *BAIAP2-AS1*, *TSIX*, and *LINC01215* has a nonsignificant negative correlation with the survival rate of GC patients. ROC analysis revealed that *BAIAP2-AS1*, *LINC01215*, and *TSIX* could have a significant role as potential diagnostic biomarkers of GC.

Furthermore, we demonstrate that the expression level of *THBS2* has a significant positive correlation with the expression of *BAIAP2-AS1*, *TSIX*, and *LINC01215*, based on the qRT-PCR experiment. This result could validate our bioinformatics coexpression analyses. Also, based on our bioinformatics analyses, miR-4677-5p has a potential interaction with *THBS2* mRNA. Low expression of *THBS2* via miR-4677-5p could be considered a potential therapeutic method for GC patients.

Previous studies demonstrated possible roles of noncoding RNAs in different patients, including multiple sclerosis [29], breast cancer [30, 31], and gastric cancer [32]. Previous studies revealed some novel information about the possible roles of *BAIAP2-AS1* in different cancer types. For example, Yang et al. in 2021 revealed that *BAIAP2-AS1* might have a regulatory role in the miR-361-3p/*SOX4* competitive endogenous RNA (ceRNA) axis. Based on this study, mentioned ceRNA axis could regulate the malignant progression of hepatocellular carcinoma (HCC). The mentioned study suggests that *BAIAP2-AS1* has a significantly high expression in HCC samples in the TCGA RNAseq datasets, qRT-PCR experiment, and HCC cell lines [33]. Mao et al. in 2018 revealed that *BAIAP2-AS1* could have a significant role in the prediction of cervical cancer survival. In the mentioned study, a high-throughput TCGA data analysis was performed to evaluate the expression level of lncRNAs and the relation of selected DEGs with the survival rate of cervical cancer patients. Based on ROC analysis, *BAIAP2-AS1* could act as a diagnostic biomarker of cervical cancer [34]. Gong et al. in 2016 revealed that *BAIAP2-AS1* has a significant upregulation in the hepatitis B virus-related HCC. Also, by silencing *BAIAP2-AS1* (using small interfering RNAs (siRNAs)), it is demonstrated that *BAIAP2-AS1* has a significant role in the regulation of *MAPKAP1* and *RAF1*, and this lncRNA could act as a ceRNA in the HCC patients [35]. There was no study about the possible role of *BAIAP2-AS1* in GC, and we performed this study of *BAIAP2-AS1* for the first time.

Previous studies revealed the possible roles of *LINC01215* in the progression of different cancers. For example, according to Liu et al. in 2020, *LINC01215* has a significant role in the survival rate of breast cancer patients (HR: 0.84, *p* value: 0.0001). Furthermore, based on the mentioned study, *LINC01215* has a significant association with immune-related functions (the result of the Pearson correlation method) [36]. Liu et al. in 2021 revealed that *LINC01215* could promote lymph node metastasis and epithelial-mesenchymal transition in ovarian cancer. Based on this study, the downregulation of *LINC01215* increases the expression level of *RUNX3* through methylation of the *RUNX3* promoter [37].

Furthermore, the downregulation of *LINC01215* suppresses tumor growth, migration, and cell proliferation of ovarian cancer [37]. Xu et al. in 2020 revealed that *LINC01215* suppresses the growth of clear cell renal cell carcinoma tumors through reducing SLC2A3 expression level via miR-184 [38]. There was no previous study about the possible role of *LINC01215* in the development of gastric cancer. About the possible role of lncRNA *TSIX* in the development of GC, Sun et al. in 2021 revealed that *TSIX* might regulate the GC development via miR-320a/*RAD51* ceRNA axis. Furthermore, this study revealed that the low expression of *TSIX* is one of the possible causes of RAD51 downregulation in the mentioned ceRNA axis. This ceRNA network simultaneously triggered the ATF6 signaling pathway following endoplasmic reticulum stress to encourage the death of GC cells and stop the illness. The *TSIX*/miR-320a/*Rad51* network offers a novel method for treating GAC disease and may be a possible biological target of the GC [39].

Our study demonstrates comprehensive novel results about the possible roles of different coding and noncoding RNAs in GC development. Based on our investigation, lncRNAs *BAIAP2-AS1*, *LINC01215*, and *TSIX* could be considered novel potential diagnostic biomarkers of GC. Based on our bioinformatics and experimental analyses, mentioned lncRNAs might regulate the expression level of *THBS2*, a high-expressed mRNA, in GC patients. *THBS2* and its interactome regulate the ECM-receptor interaction signaling pathway. Previous studies approved the possible role of the ECM-receptor signaling pathway in the regulation of progression, survival rate, and tumorigenesis of GC [28].

In our study, we introduced a potential signature model for gastric cancer survival prediction, including THBS2, BAIAP2-AS1, LINC01215, and TSIX. However, our result was not statistically significant. Based on the obtained gene score, we suggest that same investigation through different methods be evaluated on our 4 evaluated genes. Previous studies revealed some potential signature models in GC. For example, Cheong et al. at 2020 demonstrated a predictive 32-gene signature model for GC using a predictive model based on support vector machine (SVM) [40]. Another study at 2023 found a 7-gene signature model including the following genes: *CCDC91*, *DYNC1I1*, *FAM83D*, *LBH*, *SLITRK5*, *WTIP*, and *NAP1L3*. Through a similar approach to our investigation, they demonstrated that their suggested signature model modulated the TGF-beta signaling pathway [41]. Zhang et al. at 2021 introduced a 4-gene prognostic model for GC through a multivariable Cox regression analysis. Based on this article, the following four genes could have potential implications for the prediction of GC: *UTRN*, *MUC16*, *CCDC178*, and *HYDIN* [42]. However, there is no certain prediction model for GC, and more studies and validations are needed. It is highly recommended that the expression level of miR-4677-5p be evaluated by different experimental methods, like qRT-PCR. lncRNA-mRNA and miRNA-mRNA interactions in this study should be validated using different methods, like luciferase assay. A possible correlation of SNPs in the *THBS2* sequence with the binding affinity of miR-4677-5p might be a perfect method to find more accurate information about the reasons for the high or low expression of *THBS2*. Since the result of our survival analysis was not significant, we highly recommend that the possible correlation of mentioned RNAs with the survival rate of GC patients be evaluated through a bigger sample size.

## 5. Conclusion

In this study, we demonstrate that upregulation of *THBS2*, lncRNAs *BAIAP2-AS1*, *LINC01215*, and *TSIX* has a significant correlation with GC. Furthermore, for the first time, we show that lncRNAs *BAIAP2-AS1*, *LINC01215*, *TSIX*, and miR-4677-5p might regulate the expression level of *THBS2*, and any disturbance in this regulatory network might disturb the ECM-receptor interaction signaling pathway and lead the normal gastric cells to malignancy. Mentioned lncRNAs could be considered as the potential diagnostic biomarkers of GC. Also, the expression level of *THBS2*, lncRNAs *BAIAP2-AS1*, *LINC01215*, and *TSIX* might have a meaningful negative correlation with the survival rate of GC patients.

## Figures and Tables

**Table 1:** Clinicopathological analysis of gastric cancer samples.

Variable	Status	Number	%
Age	<50	8	32
	>50	17	68

Sex	Male	18	72
	Female	7	28

Tumor size	<5 cm	10	40
	>5 cm	15	60

Histology	Adenocarcinoma	23	92
	Mucinous adenocarcinoma	1	4
	Signet ring carcinoma	1	4

Perineural invasion	No	6	24
	Yes	19	76

Nodal extension	No	21	84
	Yes	4	16

TNM staging	I	1	4
	II	6	24
	IIIA	2	8
	IIIB	4	16
	IV	12	48

Family history	No	19	76
	Yes	6	24

Smoking	DX-smoker at diagnosis but discontinued	2	8
	Ex-smoker	2	8
	Nonsmoker	20	80
	smoker	1	4

**Table 2:** Table of primer sequence.

Gene	5′-->3	Forward/reverse
*THBS2*	CGTGGACAATGACCTTGTTG	F
	GCCATCGTTGTCATCATCAG	R

*BAIAP2-AS1*	ACCAGAAAGTTCCAGAGCGG	F
	ACCATGCGGATAGCTTCACC	R

*TSIX*	GTGATCCTCACAGGACTGCAACA	F
	AGCTGAGTCTTCAGCAGGTCCAA	R

*LINC01215*	AGCGCTTACCACTGTCCATT	F
	TGCCCAGGTGAACTGTTTTCT	R

*GAPDH*	AGCCACATCGCTCAGACAC	F
	GCCCAATACGACCAAATCC	R

**Table 3:** List of top 20 upregulated and downregulated genes in GSE54129.

ID	logFC	AveExpr	*p* value	Adj. *p* value	Gene symbol	Down/up
220191_at	-5.96862787	9.308647992	3.84*E*‐09	1.61*E*‐08	*GKN1*	Down
238222_at	-5.882742555	9.145683068	1.24*E*‐10	6.32*E*‐10	*GKN2*	Down
208138_at	-5.451885409	6.962448568	1.26*E*‐23	3.58*E*‐22	*GAST*	Down
205979_at	-5.275111089	5.955695606	4.36*E*‐23	1.15*E*‐21	*SCGB2A1*	Down
213921_at	-5.271075203	7.443574371	2.61*E*‐15	2.43*E*‐14	*SST*	Down
231646_at	-5.100492447	7.808510962	2.43*E*‐14	2.00*E*‐13	*DPCR1*	Down
210065_s_at	-4.868614646	7.11555797	3.38*E*‐22	7.86*E*‐21	*UPK1B*	Down
213953_at	-4.865974875	8.113243682	3.58*E*‐11	1.97*E*‐10	*KRT20*	Down
221122_at	-4.85342997	6.729619568	3.70*E*‐24	1.13*E*‐22	*HRASLS2*	Down
241137_at	-4.717247165	7.873223023	1.58*E*‐13	1.17*E*‐12	*DPCR1*	Down
243764_at	-4.688944304	8.296975061	2.35*E*‐12	1.49*E*‐11	*VSIG1*	Down
204260_at	-4.65753578	5.269594917	6.42*E*‐25	2.18*E*‐23	*CHGB*	Down
207033_at	-4.657105699	6.371562909	2.44*E*‐09	1.06*E*‐08	*GIF*	Down
234780_at	-4.632035685	4.37024875	2.54*E*‐51	2.28*E*‐48		Down
207249_s_at	-4.5518883	6.956546129	6.48*E*‐17	7.49*E*‐16	*SLC28A2*	Down
214046_at	-4.478426687	5.933183333	3.66*E*‐14	2.94*E*‐13	*FUT9*	Down
234632_x_at	-4.460705311	5.982485447	2.22*E*‐20	4.07*E*‐19		Down
210641_at	-4.440274593	6.507433205	1.54*E*‐25	5.58*E*‐24	*CAPN9*	Down
227306_at	-4.421510073	6.865494508	3.82*E*‐24	1.17*E*‐22	*RP11-363E7.4*	Down
214385_s_at	-4.329929907	8.909946864	6.51*E*‐11	3.46*E*‐10	*MUC5AC*	Down
209875_s_at	4.055061344	8.400801758	3.12*E*‐15	2.87*E*‐14	*SPP1*	Up
210764_s_at	4.074797407	8.451531992	1.01*E*‐30	7.06*E*‐29	*CYR61*	Up
203649_s_at	4.079081377	8.611325803	4.83*E*‐16	4.96*E*‐15	*PLA2G2A*	Up
209156_s_at	4.11512948	9.774724742	1.31*E*‐33	1.31*E*‐31	*COL6A2*	Up
202859_x_at	4.117296435	8.163282288	3.98*E*‐15	3.62*E*‐14	*CXCL8*	Up
218469_at	4.168860215	9.899021644	2.25*E*‐21	4.68*E*‐20	*GREM1*	Up
224646_x_at	4.199296712	7.476765447	1.15*E*‐15	1.12*E*‐14	*H19*	Up
204051_s_at	4.252115166	7.362223727	1.71*E*‐18	2.45*E*‐17	*SFRP4*	Up
202310_s_at	4.300071432	11.33718549	9.54*E*‐36	1.28*E*‐33	*COL1A1*	Up
201289_at	4.344681412	9.535824144	9.94*E*‐36	1.33*E*‐33	*CYR61*	Up
1555229_a_at	4.398480865	8.495294055	5.14*E*‐39	1.15*E*‐36	*C1S*	Up
209395_at	4.412985552	7.207506621	4.45*E*‐17	5.27*E*‐16	*CHI3L1*	Up
223121_s_at	4.462926494	8.277239652	1.74*E*‐19	2.83*E*‐18	*SFRP2*	Up
201058_s_at	4.469669609	10.22080471	1.82*E*‐24	5.81*E*‐23	*MYL9*	Up
218468_s_at	4.580281615	9.473598068	6.43*E*‐24	1.90*E*‐22	*GREM1*	Up
227140_at	4.681301515	7.462719811	8.61*E*‐27	3.71*E*‐25	*INHBA*	Up
226237_at	4.766250328	7.943784326	2.30*E*‐26	9.38*E*‐25	*COL8A1*	Up
238320_at	4.893182068	8.962860189	5.35*E*‐50	4.31*E*‐47	*MIR612*	Up
223122_s_at	5.876935726	9.798533583	1.31*E*‐26	5.49*E*‐25	*SFRP2*	Up
227404_s_at	5.901635278	10.46172328	3.38*E*‐60	8.45*E*‐57	*EGR1*	Up

**Table 4:** List of significant upregulated genes in the ECM-receptor interaction pathway.

Symbol	Rank in gene list	Rank metric score	Running ES
*THBS2*	13	1.38	0.0489
*SPP1*	17	1.317	0.0962
*COL6A2*	28	1.222	0.1396
*ITGA5*	54	1.079	0.1771
*THBS4*	62	1.045	0.2143
*THBS1*	130	0.86	0.2417
*COMP*	166	0.811	0.269
*COL11A1*	204	0.764	0.2945
*ITGA7*	212	0.753	0.3212
*FN1*	224	0.739	0.3472
*TNC*	256	0.703	0.3709
*COL4A4*	315	0.663	0.3917
*COL6A3*	367	0.629	0.4116
*COL4A1*	561	0.537	0.4208
*COL1A2*	741	0.481	0.4287
*ITGA11*	751	0.479	0.4455
*COL5A3*	765	0.476	0.4619
*COL4A2*	842	0.457	0.4743
*COL5A1*	863	0.451	0.4895
*VWF*	907	0.443	0.5032
*SV2A*	909	0.443	0.5191
*COL5A2*	1021	0.421	0.5284
*LAMA5*	1058	0.414	0.5414
*SDC3*	1162	0.396	0.5502
*COL6A1*	1174	0.393	0.5638
*LAMA4*	1290	0.374	0.5712
*HSPG2*	1382	0.361	0.5793
*LAMC1*	1600	0.332	0.5799
*ITGA1*	1713	0.319	0.5855
*SV2B*	1770	0.313	0.5938
*TNR*	1793	0.31	0.6038
*COL1A1*	1813	0.307	0.6138
*COL3A1*	1995	0.285	0.6146
*LAMA2*	2304	0.257	0.6076
*THBS3*	2416	0.248	0.6107
*RELN*	2423	0.248	0.6193
*CD44*	2458	0.245	0.6264
*IBSP*	2731	0.225	0.6202
*TNN*	2747	0.225	0.6275

**Table 5:** Gene ontology analysis of *THBS2* and related proteins, based on enrichr database.

Gene ontology		
Term	Adjusted *p* value	Genes
*Cellular component*		
Collagen-containing extracellular matrix (GO:0062023)	0.028067617	*ADAMTS1*, *MMP2*, *THBS2*

*Molecular function*		
Metalloendopeptidase activity (GO:0004222)	4.30*E*‐11	*ADAMTSL1*, *ADAMTS5*, *ADAMTS2*, *ADAMTS1*, *MMP2*, *ADAMTS12*
Metallopeptidase activity (GO:0008237)	2.34*E*‐10	*ADAMTSL1*, *ADAMTS5*, *ADAMTS2*, *ADAMTS1*, *MMP2*, *ADAMTS12*
Endopeptidase activity (GO:0004175)	5.03*E*‐08	*ADAMTSL1*, *ADAMTS5*, *ADAMTS2*, *ADAMTS1*, *MMP2*, *ADAMTS12*
C-X3-C chemokine binding (GO:0019960)	0.013186586	*ITGB1*
Collagen binding involved in cell-matrix adhesion (GO:0098639)	0.013186586	*ITGB1*
Cell-matrix adhesion mediator activity (GO:0098634)	0.015376688	*ITGB1*
Clathrin heavy chain binding (GO:0032050)	0.016462703	*LRP1*
Protein binding involved in heterotypic cell-cell adhesion (GO:0086080)	0.016462703	*CD47*
Cell adhesion mediator activity (GO:0098631)	0.034997935	*CD47*
Lipoprotein particle receptor binding (GO:0070325)	0.036711163	*LRP1*
Chemokine binding (GO:0019956)	0.038103413	*ITGB1*
Cell-cell adhesion mediator activity (GO:0098632)	0.045728931	*CD47*

*Biological process*		
Extracellular structure organization (GO:0043062)	3.00*E*‐12	*ITGB1*, *ADAMTSL1*, *ADAMTS5*, *ADAMTS2*, *ADAMTS1*, *MMP2*, *CD47*, *ADAMTS12*
External encapsulating structure organization (GO:0045229)	3.00*E*‐12	*ITGB1*, *ADAMTSL1*, *ADAMTS5*, *ADAMTS2*, *ADAMTS1*, *MMP2*, *CD47*, *ADAMTS12*
Extracellular matrix organization (GO:0030198)	2.74*E*‐11	*ITGB1*, *ADAMTSL1*, *ADAMTS5*, *ADAMTS2*, *ADAMTS1*, *MMP2*, *CD47*, *ADAMTS12*
Integrin-mediated signaling pathway (GO:0007229)	4.54*E*‐04	*ITGB1*, *ADAMTS1*, *CD47*
Positive regulation of vascular-associated smooth muscle cell proliferation (GO:1904707)	0.002549534	*ADAMTS1*, *MMP2*
Cellular response to cytokine stimulus (GO:0071345)	0.003555608	*ITGB1*, *MMP2*, *CD47*, *ADAMTS12*
Regulation of angiogenesis (GO:0045765)	0.005029385	*ITGB1*, *ADAMTS1*, *THBS2*
Regulation of vascular-associated smooth muscle cell proliferation (GO:1904705)	0.005029385	*ADAMTS1*, *MMP2*

**Table 6:** Pathway enrichment analysis of *THBS2* and correlated proteins.

Term	Adjusted *p* value	Genes
ECM-receptor interaction	4.63*E*‐04	*ITGB1*, *CD47*, *THBS2*
Malaria	0.005811118	*LRP1*, *THBS2*
Leukocyte transendothelial migration	0.019982511	*ITGB1*, *MMP2*
Phagosome	0.02639964	*ITGB1*, *THBS2*
Focal adhesion	0.031562049	*ITGB1*, *THBS2*
Proteoglycans in cancer	0.031562049	*ITGB1*, *MMP2*

## Data Availability

The datasets generated or analyzed during the current study are available in the GEO repository, GSE54129.
